# Exposure to RF-EMF Alters Postsynaptic Structure and Hinders Neurite Outgrowth in Developing Hippocampal Neurons of Early Postnatal Mice

**DOI:** 10.3390/ijms22105340

**Published:** 2021-05-19

**Authors:** Ju Hwan Kim, Kyung Hwun Chung, Yeong Ran Hwang, Hye Ran Park, Hee Jung Kim, Hyung-Gun Kim, Hak Rim Kim

**Affiliations:** 1 Department of Pharmacology, College of Medicine, Dankook University, Cheonan 31116, Korea; jhkim731@dankook.ac.kr (J.H.K.); phr1355@gmail.com (H.R.P.); hgkimm@dankook.ac.kr (H.-G.K.); 2 Hyangseol Medical Research Center, Soonchunhyang University Bucheon Hospital, Bucheon 14584, Korea; cyung@hanmail.net; 3 Department of Physiology, College of Medicine, Dankook University, Cheonan 31116, Korea; wcl6231@naver.com (Y.R.H.); heejungkim@dankook.ac.kr (H.J.K.)

**Keywords:** RF-EMF, hippocampus, dendritic spine, neurite outgrowth, glutamate receptor, neuron

## Abstract

Exposure to radiofrequency electromagnetic fields (RF-EMFs) has increased rapidly in children, but information on the effects of RF-EMF exposure to the central nervous system in children is limited. In this study, pups and dams were exposed to whole-body RF-EMF at 4.0 W/kg specific absorption rate (SAR) for 5 h per day for 4 weeks (from postnatal day (P) 1 to P28). The effects of RF-EMF exposure on neurons were evaluated by using both pups’ hippocampus and primary cultured hippocampal neurons. The total number of dendritic spines showed statistically significant decreases in the dentate gyrus (DG) but was not altered in the cornu ammonis (CA1) in hippocampal neurons. In particular, the number of mushroom-type dendritic spines showed statistically significant decreases in the CA1 and DG. The expression of glutamate receptors was decreased in mushroom-type dendritic spines in the CA1 and DG of hippocampal neurons following RF-EMF exposure. The expression of brain-derived neurotrophic factor (BDNF) in the CA1 and DG was significantly lower statistically in RF-EMF-exposed mice. The number of post-synaptic density protein 95 (PSD95) puncta gradually increased over time but was significantly decreased statistically at days in vitro (DIV) 5, 7, and 9 following RF-EMF exposure. Decreased BDNF expression was restricted to the soma and was not observed in neurites of hippocampal neurons following RF-EMF exposure. The length of neurite outgrowth and number of branches showed statistically significant decreases, but no changes in the soma size of hippocampal neurons were observed. Further, the memory index showed statistically significant decreases in RF-EMF-exposed mice, suggesting that decreased synaptic density following RF-EMF exposure at early developmental stages may affect memory function. Collectively, these data suggest that hindered neuronal outgrowth following RF-EMF exposure may decrease overall synaptic density during early neurite development of hippocampal neurons.

## 1. Introduction

Exposure to radiofrequency electromagnetic fields (RF-EMFs) has become unavoidable due to the widespread use of wireless communication devices in modern society. As mobile phone use typically requires close contact with the head, possible effects on the brain are of particular concern [1]. Despite various controversies, there is accumulating evidence regarding the biological effects of RF-EMF exposure on the central nervous system (CNS), such as changes in intracellular calcium homeostasis, neuronal damage, and perturbations in neurotransmitter systems [2,3,4].

In particular, exposure to RF-EMF has increased rapidly in children. Mobile phone use has become the major determinant of RF-EMF exposure [5]. Recent studies have demonstrated that the specific absorption rate (SAR) of 5-year-old children is 1.5-fold higher than that of 20-year-old adults [6,7]. Therefore, RF-EMF exposure may have a greater impact on children during the developmental stages of the nervous system. We recently demonstrated that exposure to 1850 MHz RF-EMF in early postnatal mice resulted in morphological changes in synapses in the auditory brainstem in the absence of functional abnormalities [8].

The hippocampus is a component of the limbic system and plays a decisive role in the formation of spatial navigation and new memories, and is crucial for emotion and learning [9,10,11]. Altered functional connectivity in the hippocampus is strongly implicated in early Alzheimer’s disease [12,13], and hippocampal atrophy is associated with mental disorders, such as schizophrenia and depressive disorders [14,15]. In addition, activity-dependent modification of synaptic connections modulates the ability of the nervous system to adapt, learn, and form memories [16].

Synapses are essential for neuronal function. Individual neurons pass chemical and/or electrical signals to other neurons in the nervous system [17]. We previously demonstrated that synaptic vesicles in the presynaptic axonal terminal were altered following RF-EMF exposure in mice [8,18,19,20]. Generally, electrical activity in the axon terminal of a presynaptic neuron is converted into the release of neurotransmitters that bind to receptors located on the dendrites of postsynaptic cells. The release of neurotransmitters from the axon terminal determines neuronal function [21].

Dendritic spines (DSs) are small cytoplasmic extensions of dendrites that form the postsynaptic compartment of the majority of excitatory synapses in the mammalian brain. Alterations in the density, size, and shape of DSs (e.g., mushroom, stubby, and thin) are correlated with neuronal dysfunction; thus, their structure and density are crucial determinants of neuronal input–output transformations [22]. In addition, DSs provide an anatomical substrate for memory storage and synaptic transmission in the brain. Indeed, gain, loss, and morphological remodeling of DSs are involved in learning and memory [23].

The surface of DSs contains glutamate receptors known as α-amino-3-hydroxy5-methyl-4-isoxazolepropionic acid (AMPA) and *N*-methyl-*D*-aspartate (NMDA) receptors, which play important roles in synaptic plasticity and are considered vital for memory and learning [24,25]. Additionally, brain-derived neurotrophic factor (BDNF) stimulates dendritic growth and increases synaptic density during dendrite development [26,27,28,29].

Nevertheless, it remains unclear whether RF-EMF affects synapse formation and neurite outgrowth in hippocampal neurons during neuronal development. Therefore, in the present study, changes in dendritic formation in hippocampal neurons during developing stages of the nervous system were examined following the exposure of mice to 1850 MHz RF-EMF at 4.0 W/kg SAR (5 h daily for 4 weeks). We also investigated the effects of RF-EMF exposure on hippocampal synapse formation and neuronal outgrowth. The physiological effects of these changes were tested using a neurobehavioral test for memory.

## 2. Results

### 2.1. Exposure to RF-EMF Decreases the Number of DSs in Hippocampal Neurons

**Figure 1:**
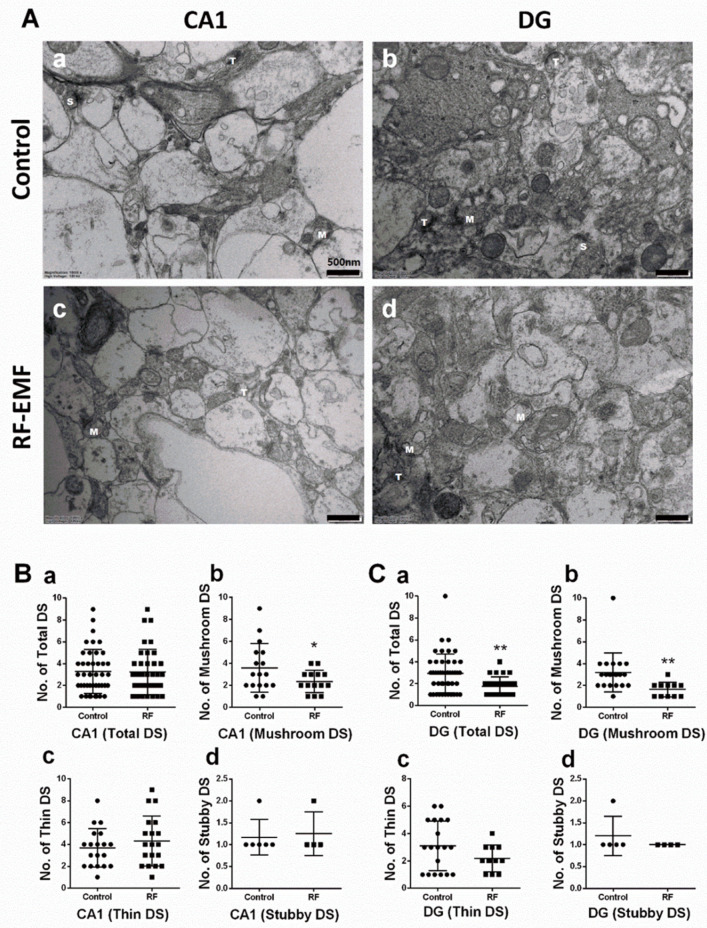
Changes in dendritic spines in hippocampal CA1 and DG of RF-EMF-exposed early postnatal mice. (**A**). Representative TEM images of dendritic spines of hippocampal CA1 (**a**,**c**) and DG (**b**,**d**) in control (**a**,**b**) and RF-EMF-exposed mice (**c**,**d**). The number of thin (**b**), stubby (**c**), and mushroom-type (**d**) dendritic spines and the total number of dendritic spines (**a**) were analyzed in hippocampal CA1 (**B**) and DG (**C**). Data are expressed as means ± SD. Statistical significance was evaluated using the Student’s *t*-test. * *p* < 0.05, ** *p* < 0.01 vs. control (control *n* = 4 mice, RF-EMF *n* = 4 mice). M, mushroom; T, thin; S, Stubby; ● Control; ■ RF-EMF. Scale bars = 500 nm.

DSs of neurites in the hippocampus were analyzed using hippocampal sections isolated from mice exposed to 1850 MHz RF-EMF at a SAR value of 4.0 W/kg for 5 h per day for 4 weeks to examine the effects of RF-EMF exposure on neurodevelopment and synapse formation. The total number of DSs showed a statistically significant decrease in the dentate gyrus (DG) (Cohen’s d = 0.77) but was not altered in the cornu ammonis (CA1). DSs can be classified into three types (mushroom, thin, and stubby; Figure S1). Additional analysis was performed for each subtype in both CA1 and DG. The number of mushroom-type DSs showed statistically significant decreases in both CA1 (Cohen’s d = 0.71) and DG (Cohen’s d = 1.03) (Figure 1Bb,Cb), but the number of stubby and thin-type DSs was not changed (Figure 1).

### 2.2. Glutamate Receptor Expression in Hippocampal Neurons Is Decreased Following RF-EMF Exposure

**Figure 2:**
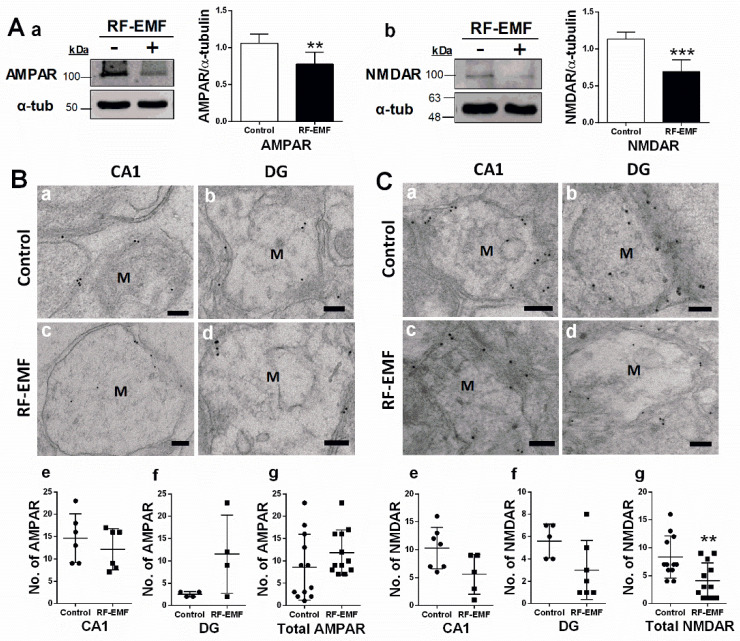
Expression levels of AMPAR and NMDAR in the hippocampus of RF-EMF-exposed early postnatal mice. (**A**). Protein expression levels of AMPAR (control *n* = 7 mice, RF-EMF *n* = 7 mice) and NMDAR (control *n* = 6 mice, RF-EMF *n* = 6 mice) in mouse hippocampus. Total protein was immunoblotted with antibodies against AMPAR (**a**) and NMDAR (**b**). The intensities of bands were quantified by densitometry. Protein levels were normalized relative to α-tubulin. (**B**,**C**). Representative immunogold staining for glutamate receptors, AMPAR (**B**) and NMDAR (**C**), in dendritic spines of hippocampal CA1 (**a**,**c**) and DG (**b**,**d**) in control (**a**,**b**) and RF-EMF-exposed mice (**c**,**d**). Gold particles labeling AMPAR and NMDAR were observed by transmission electron microscopy. The total number of gold particles labeling AMPAR (Bg) and NMDAR (Cg) in hippocampal CA1 (**e**) and DG (**f**) were quantified in mushroom-type dendritic spines. Data are expressed as means ± SD. Statistical significance was evaluated using the Student’s *t*-test. ** *p* < 0.01, *** *p* < 0.001 vs. control (control *n* = 4 mice, RF-EMF *n* = 4 mice). M, mushroom; ● Control; ■ RF-EMF. Scale bars = 200 nm.

Glutamate receptors are integral for plasticity and synaptic transmission at many postsynaptic membranes. We, therefore, analyzed the expression of AMPA and NMDA receptors in the hippocampus using immunoblots (Figure 2A) and immunogold staining (Figure 2B) in hippocampal sections. The total expression of AMPA and NMDA receptors in the hippocampus was statistically significantly decreased in the hippocampus of mice following RF-EMF exposure (Figure 2A). Due to difficulties in separating CA1 and DG due to the small size of these loci, the distribution of glutamate receptors in subareas of hippocampal neurons was examined using immunogold staining for AMPAR and NMDAR in DSs in the hippocampal CA1 and DG areas (Figure 2B). We focused on mushroom-type DSs, given that only this subtype was statistically significantly altered following RF-EMF exposure (Figure 1). The number of gold particles labeling AMPA and NMDA receptors within the membranes of mushroom-type DSs was analyzed (Figure 2). The number of gold particles labeling AMPAR1 (GluR1) and NMDAR1 (NR1) was not altered in the CA1 and DG of the hippocampus following RF-EMF exposure (Figure 2B,C). However, the total number of gold particles labeling hippocampal NMDAR1 (Cohen’s d = 1.21) statistically significant decreased following RF-EMF exposure (Figure 2C).

### 2.3. Brain-Derived Neurotrophic Factor (BDNF) Expression in Hippocampal Neurons Is Decreased Following RF-EMF Exposure

**Figure 3:**
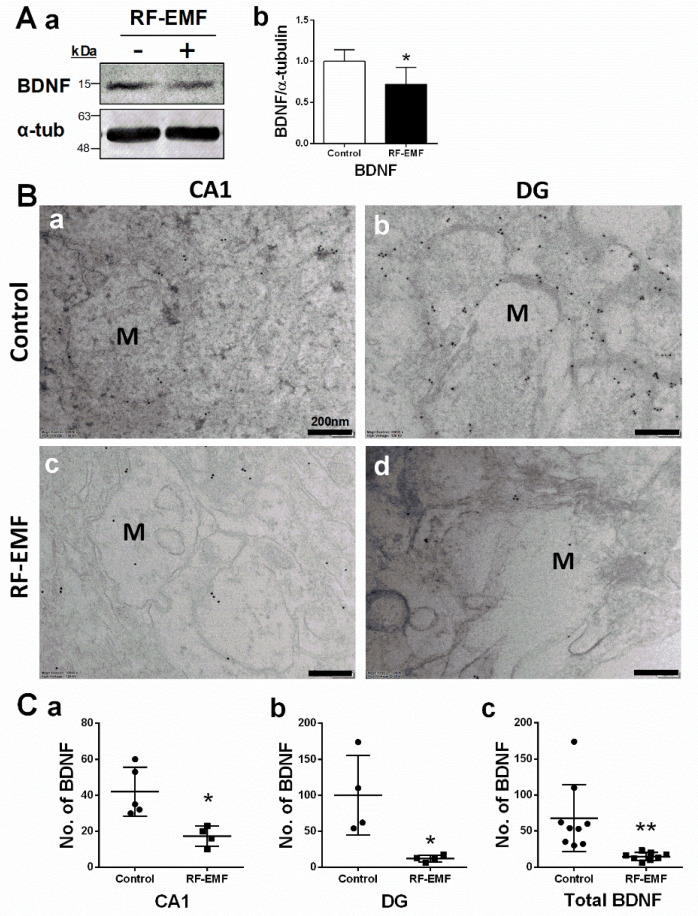
Expression levels of BDNF in the hippocampus of RF-EMF-exposed early postnatal mice. (**A**). Protein expression levels of BDNF in mouse hippocampus (control *n* = 5 mice, RF-EMF *n* = 5 mice). Total protein was immunoblotted with an antibody against BDNF (**a**). The intensities of bands were quantified by densitometry (**b**). Protein levels were normalized relative to α-tubulin. (**B**). Representative immunogold staining for BDNF in hippocampal CA1 (**a**,**c**) and DG (**b**,**d**) in control (**a**,**b**) and RF-EMF-exposed mice (**c**,**d**). Gold particles labeling BDNF were observed by transmission electron microscopy. (**C**). The total number of gold particles labeling BDNF (**c**) in hippocampal CA1 (**a**) and DG (**b**) were quantified in hippocampal neurons. Data are expressed as means ± SD. Statistical significance was evaluated using the Student’s *t*-test. * *p* < 0.05, ** *p* < 0.01 vs. control (control *n* = 4 mice, RF-EMF *n* = 4 mice). M, mushroom; ● Control; ■ RF-EMF. Scale bars = 200 nm.

BDNF is a member of the neurotrophic family of growth factors and plays an important role in synaptic survival, synapse formation, neurite outgrowth, and synaptic plasticity [26,27,28]. BDNF expression in the hippocampus was analyzed using immunoblots (Figure 3A) and immunogold staining (Figure 3B) in hippocampal sections isolated from mice exposed to 1850 MHz RF-EMF at a SAR value of 4.0 W/kg for 5 h per day for 4 weeks. BDNF expression levels showed a statistically significant decrease in the hippocampus of mice following RF-EMF exposure (Figure 3A). The distribution of BDNF in the CA1 and DG subareas of hippocampal neurons was analyzed using immunogold staining (Figure 3B). The number of gold particles labeling BDNF was quantified in the axon-spine of dendrites in hippocampal neurons of mice following RF-EMF exposure. The number of gold particles labeling BDNF showed statistically significant decreases in CA1 (Cohen’s d = 2.27) and DG (Cohen’s d = 2.25) regions following RF-EMF exposure (Figure 3C).

### 2.4. Synaptic Density in Hippocampal Neurons Is Decreased Following Exposure to RF-EMF

**Figure 4:**
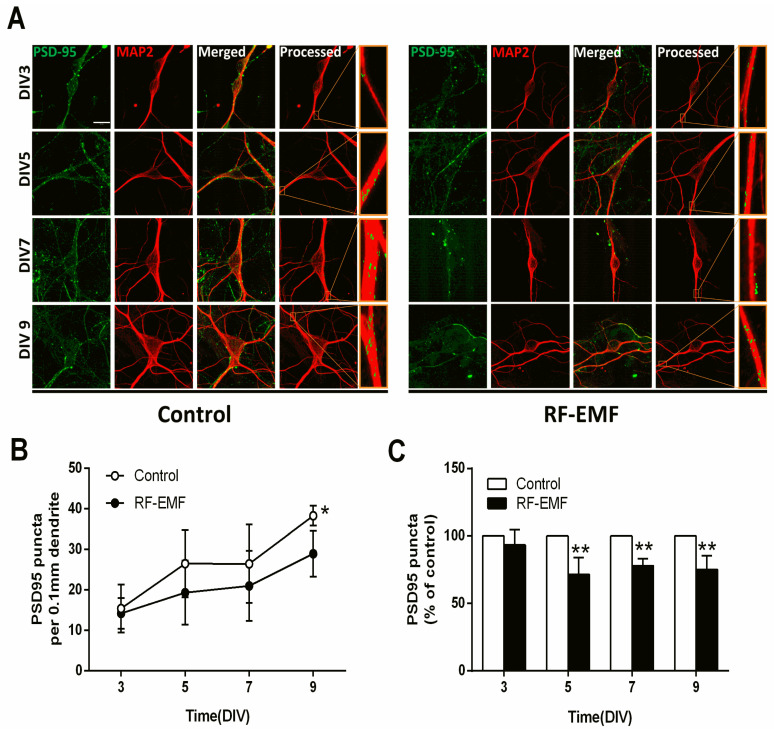
Exposure to RF-EMF decreased synaptic densities in mouse hippocampal neurons. (**A**). Confocal images show hippocampal neurons expressing PSD95 (green) and MAP2 (red). Images of PSD95 and MAP2 were analyzed using ImageJ. (**B**). Time-dependent changes in PSD95-positive punctae in dendrites of mouse hippocampal neurons. (**C**). Summary of the changes in PSD95 punctae as a percentage of control. Data are expressed as means ± SD. Statistical significance was evaluated using either ANOVA (**B**) or Student’s *t*-test (**C**). * *p* < 0.05, ** *p* < 0.01 vs. control (control *n* = 20 mice, RF-EMF *n* = 16 mice). Scale bars = 15 µm.

Hippocampal neurons were isolated from ICR mice at P1 and exposed to 1760 MHz RF-EMF at 4.0 W/kg for 5 h/day for 9 days to assess the effects of RF-EMF exposure on synaptic density during synapse development. Postsynaptic density 95 (PSD95) plays an important role in synaptic plasticity. The PSD95 puncta present in the primary dendrite derived from one neuron, namely as any branch emerging from the soma [30], was counted. Microtubule-associated protein 2 (MAP2) is a neuron-specific cytoskeletal protein that is enriched in dendrites and is used as a dendritic marker [31]. Primary cultured hippocampal neurons were stained with anti-PSD95 and anti-MAP2 antibodies on DIV 3, 5, 7, and 9 (Figure 4A). The results were illuminated using control cells and RF-EMF exposed cells in DIV3 (*n* = 9 cells), DIV5 (*n* = 12 cells), DIV7 (*n* = 12 cells) and DIV9 (*n* = 15 cells). The average number of dendrites per cell was DIV3 (control *n* = 3 ± 0; RF-EMF *n* = 2.65 ± 0.35), DIV5 (control *n* = 2.7 ± 0.2, RF-EMF *n* = 2.7 ± 0.1), DIV7 (control *n* = 2.8 ± 0.1, RF-EMF *n* = 2.5 ± 0.3), and DIV9 (control *n* = 3 ± 0, RF-EMF *n* = 3 ± 0). The distance from soma to dendrite was DIV3 (control 38.9 μm, RF-EMF 43.6 μm), DIV5 (control 57.6 μm, RF-EMF 53.0 μm), DIV7 (control 52.2 μm, RF-EMF 52.9 μm), and DIV9 (control 58.2 μm, RF-EMF 57.6 μm). The number of PSD95 puncta increased with time from DIV 3 to 9 and reached a peak on DIV 9. In the RF-EMF-exposed group, although the number of PSD95 puncta also gradually increased over time, it was significantly lower in statistical analysis on DIV 5, 7, and 9 compared to that in control cells (Figure 4B). However, the rate of reduction differed between the RF-EMF-exposed group and control group was not altered on the same DIV (Figure 4C).

### 2.5. Glutamate Receptor Expression in Primary Cultured Hippocampal Neurons Is Decreased Following RF-EMF Exposure

**Figure 5:**
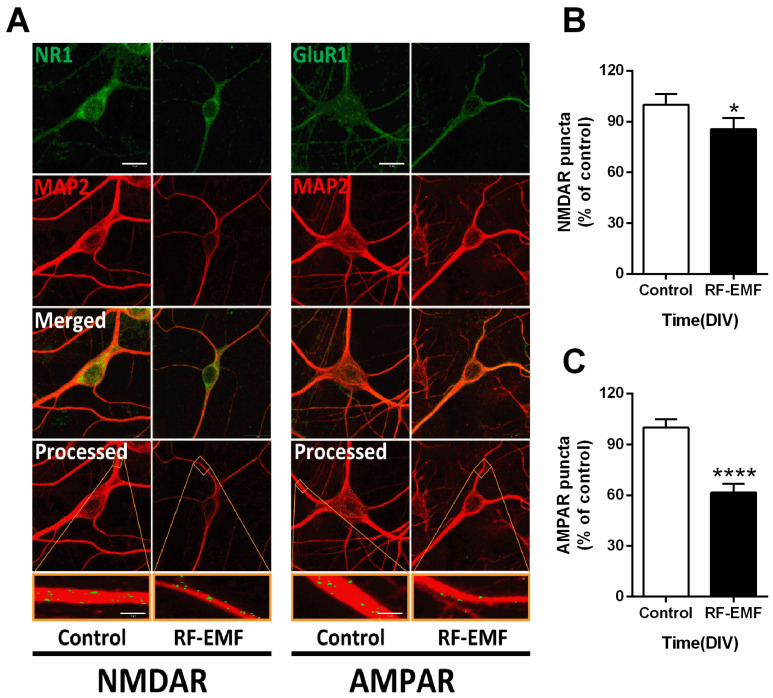
Exposure to RF-EMF decreased the expression of NMDAR and AMPAR. (**A**). Confocal images display hippocampal neurons expressing AMPAR (GluR1, green) or NMDAR (NR1, green) with MAP2 (red). Images of GluR1 and NR1 with MAP2 were analyzed using ImageJ. (**B**,**C**). Summary of changes in the number of GluR1 and NR1 punctae. Data are expressed as means ± SD. Statistical significance was evaluated using the Student’s *t*-test. * *p* < 0.05, **** *p* < 0.0001 vs. control (control *n* = 18 mice, RF-EMF *n* = 18 mice). Scale bars = 15 µm.

Given the decrease in synaptic density in RF-EMF-exposed mice, we further examined the expression levels of glutamate receptors (NMDA and AMPA receptors), which are involved in synapse formation, in primary cultured hippocampal neurons on DIV 9. The number of cells counted was the NR1 (control *n* = 12 cells, RF-EMF *n* = 12 cells) and the GluR1 (control *n* = 12 cells, RF-EMF *n* = 12 cells). The average number of dendrites per cell was NR1 (control *n* = 2.8 ± 0.1, RF-EMF *n* = 3 ± 0) and the GluR1 (control *n* = 2.5 ± 0.2, RF-EMF *n* = 2.7 ± 0.1). NMDAR and AMPAR at dendrites were immunostained with antibodies specific to NMDAR1 (NR1) and AMPAR1 (GluR1), respectively (Figure 5A). The number of punctae labeled for AMPAR1 (GluR1) and NMDAR1 (NR1) showed statistically significant decreases following RF-EMF exposure in primary cultured hippocampal neurons (Figure 5B,C). In in vivo experiments, NMDAR expression was affected following RF-EMF (Figure 2); this effect was even greater for AMPAR in the cell culture model (Figure 5).

### 2.6. BDNF Expression Is Decreased at the Soma but Not Neurites of Hippocampal Neurons Following RF-EMF Exposure

**Figure 6:**
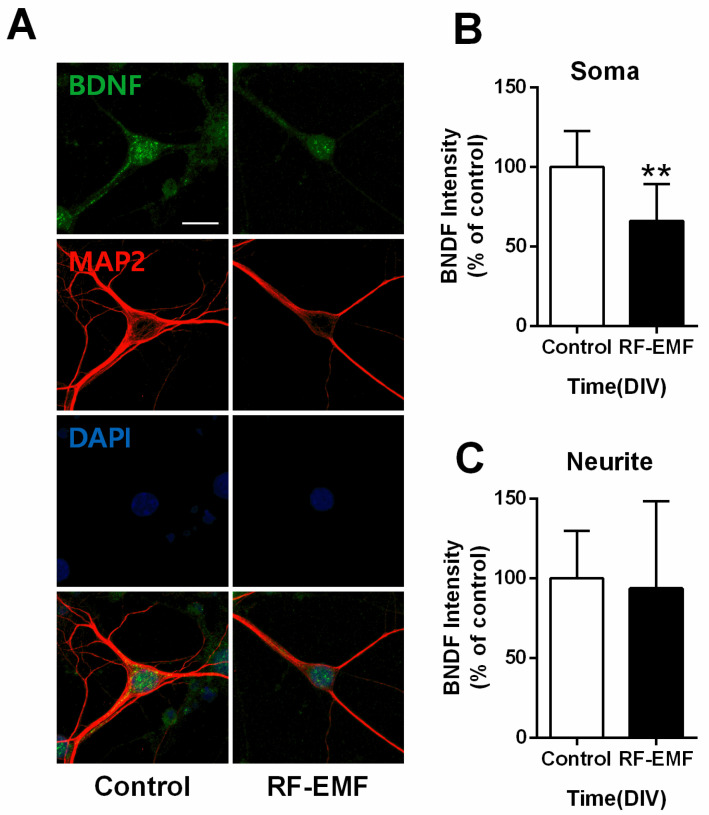
Exposure to RF-EMF decreased somatic BDNF expression in primary cultured hippocampal neurons. (**A**). Confocal images display hippocampal neurons expressing BDNF (green), MAP2 (red), and nuclei (DAPI, blue). Expression of BDNF in hippocampal neurons was analyzed on DIV 9. (**B**,**C**). Summary of changes in BDNF expression in the soma and neurites of cultured hippocampal neurons. Data are expressed as means ± SD. Statistical significance was evaluated using the Student’s *t*-test. ** *p* < 0.01 vs. control (control *n* = 18 mice, RF-EMF *n* = 18 mice). Scale bar = 15 µm.

Given the decrease in BDNF expression in RF-EMF-exposed mice (Figure 3), we further examined expression levels of BDNF, a neurotrophic factor involved in synaptogenesis [32], in primary cultured hippocampal neurons on DIV 9. Neurites of hippocampal neurons were identified using MAP2 immunostaining. In addition, BDNF level in the soma and neurite was as follows (control *n* = 9 cells, RF-EMF *n* = 9 cells). The average number of dendrites per cell was as follows (control *n* = 3.64 ± 0.5, RF-EMF *n* = 3.5 ± 0.36). BDNF expression levels showed statistically significant decreases only at the soma (Figure 6B), but no changes were noted in the neurites of hippocampal neurons (Figure 6C).

### 2.7. Neurite Outgrowth of Hippocampal Neurons Is Affected by RF-EMF Exposure

**Figure 7:**
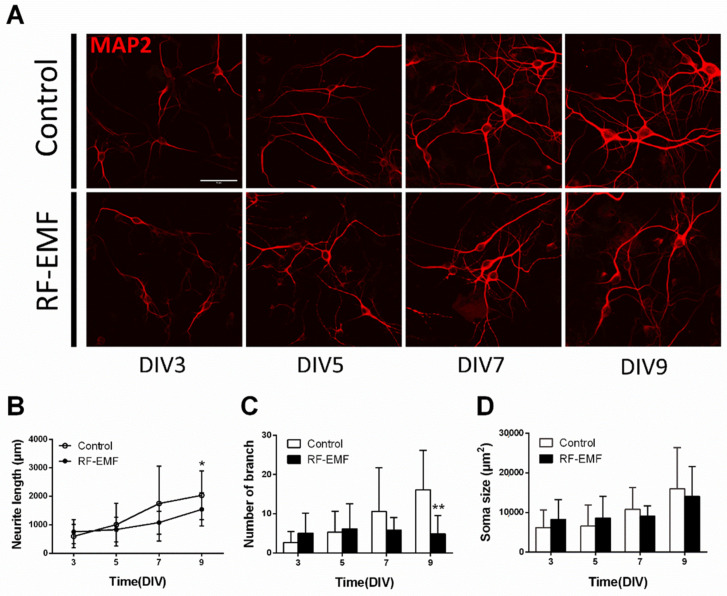
Exposure to RF-EMF decreased neurite outgrowth in developing hippocampal neurons. (**A**). Confocal images display neurite outgrowth (MAP2, red) in developing neurons. (**B**). Time-dependent changes in neurite length C. Number of branches D. Soma size. Data are expressed as means ± SD. Statistical significance was evaluated using ANOVA (**B**) or Student’s *t*-test (**C**,**D**). * *p* < 0.05, ** *p* < 0.01 vs. control (control *n* = 20 mice, RF-EMF *n* = 16 mice). Scale bar = 15 µm.

Given the decrease in synaptic density in primary cultured hippocampal neurons following RF-EMF exposure (Figure 4), and we further investigated whether neurite outgrowth of cultured hippocampal neurons was affected during synaptic development following RF-EMF exposure. Neurite outgrowth was analyzed by measuring neurite length (µm), branch number, and soma size (µm^2^) after immunostaining for MAP2 (Figure 7A). The number of cells counted was DIV3 (control *n* = 12 cells, RF-EMF *n* = 7 cells), DIV5 (control *n* = 11 cells, RF-EMF *n* = 8 cells), DIV7 (control *n* = 11 cells, RF-EMF *n* = 6 cells), and DIV9 (control *n* = 13 cells, RF-EMF *n* = 8 cells). The average number of dendrites per cell was DIV3 (control *n* = 2.5 ± 0.5; RF-EMF *n* = 2.3 ± 0.05), DIV5 (control *n* = 2.6 ± 0.2, RF-EMF *n* = 2.5 ± 0.24), DIV7 (control *n* = 3.3 ± 0.13, RF-EMF *n* = 2.6 ± 0.37), and DIV9 (control *n* = 3.5 ± 0.3, RF-EMF *n* = 2.7 ± 0.06).

Neurite length increased with time from DIV 3 to DIV 9 and reached a peak on DIV 9. In the RF-EMF-exposed group, neurite length also increased with time but to a lesser extent than that in control cells (Figure 7B). The number of neuronal branches increased with time in the control group but not in the RF-EMF-exposed group; indeed, the number of branches on DIV 9 showed a statistically significant decrease following RF-EMF exposure, compared to control (Figure 7C). However, given that soma size increased with time in both control and RF-EMF groups, no differences in soma size were noted (Figure 7D).

### 2.8. Memory Index and Discrimination Ratio Is Decreased Following RF-EMF Exposure

**Figure 8:**
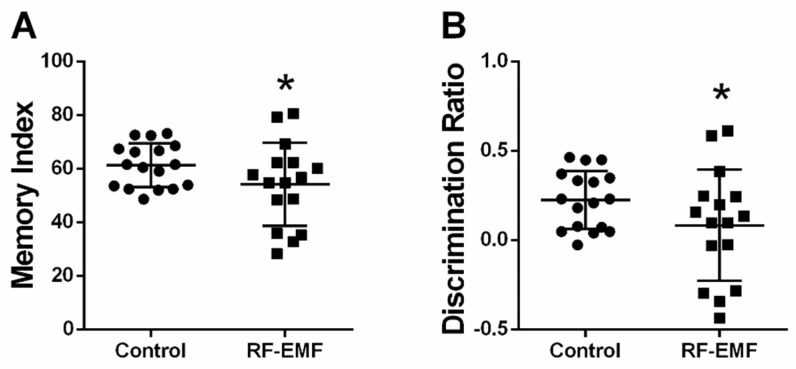
Novel object recognition test in RF-EMF-exposed early postnatal mice. Mice were exposed to 1850 MHz RF-EMF at a SAR of 4.0 W/kg for 5 h per day for 4 weeks (from P1 to P28). The memory index was decreased in RF-EMF-exposed mice (control *n* = 17 mice, RF-EMF *n* = 16 mice). The memory index was calculated by measuring interaction time with the novel object (**A**). The discrimination ratio was calculated by measuring the exploration time for the novel object compared to the old object as a proportion of the total exploration time (**B**). Data are expressed as means ± SD. Statistical significance was evaluated using the Student’s *t*-test. * *p* < 0.05.

Given the effects on synaptic densities following RF-EMF exposure at the early developmental stages of hippocampal neurons, memory function was tested using the novel object recognition test in mice after exposure to 1850 MHz RF-EMF at a SAR value of 4.0 W/kg for 5 h per day for 4 weeks. The memory index (Cohen’s d = 0.58) and discrimination ratio (Cohen’s d = 0.58) showed statistically significant decreases in RF-EMF-exposed mice, compared to control (Figure 8A,B, respectively). All Cohen’s d values are listed in Appendix A.

## 3. Discussion

In this study, we studied the possible effects of RF-EMF on the formation of DSs of postsynaptic neurons in the mouse hippocampus. DSs are small membranous protrusions from neuronal dendrites that receive excitatory input from a single axon at the synapse, which is the major site of contact for glutamatergic presynaptic inputs in the mammalian central nervous system [22]. DSs are motile and diverse in size and shape and are composed of a spine head and neck [33]. In general, large spines form proportionally large synapses [33]. The size of the spine head is proportional to the area of the postsynaptic density and is proportional to the number of postsynaptic receptors located on the postsynaptic membrane and the number of synaptic vesicles docked at the opposing presynaptic membrane [34,35]. Therefore, larger spine heads are associated with stronger synaptic signaling. Different types of DSs exist, commonly classified as thin, stubby, and mushroom-shaped (Figure S1).

Exposure to RF-EMF statistically significantly decreased the number of mushroom-type DSs in the hippocampal CA1 and DG regions of early postnatal mice, but the total number of DSs was only decreased in the DG but not CA1 region of the hippocampus (Figure 1). These results are consistent with previous reports that the number of mushroom-type DSs in rat cortex was decreased following exposure to extremely low-frequency electromagnetic field (ELF-EMF) for 14 or 28 days [36]. Alterations in the number of mushroom-type DSs of hippocampal neurons may alter the synaptic formation and induce changes in the synaptic function. Moreover, spine morphology is subject to rapid alteration by patterns of neuronal activity and postsynaptic glutamate receptor activation [37,38].

Glutamate is the most abundant neurotransmitter in the CNS and is implicated in synaptic plasticity, which underpins cognitive functions, such as learning and memory [39]. Ionotropic glutamate receptors comprise AMPARs and NMDARs, each consisting of one or more protein subunits and their associated signaling partners. These receptors are crucial regulators of synaptic plasticity in postsynaptic neurons [40]. Activation of these receptors is responsible for basal excitatory synaptic transmission and many forms of synaptic plasticity termed long-term potentiation (LTP), which is thought to underlie learning and memory [40]. The hippocampus plays an important role in learning and memory [11]. The expression levels of AMPA and NMDA receptors were statistically significantly decreased in hippocampal neurons following RF-EMF exposure. AMPA and NMDA receptor expression levels at the synapse are determined by postsynaptic size [41]. Therefore, a decrease in the total number of postsynaptic glutamate receptors following RF-EMF exposure could at least partly be caused by a decrease in the number of mushroom-type DSs with the largest heads. The distribution of active AMPA and NMDA receptors was examined using gold particle-labeled specific antibodies for glutamate receptors in mushroom-type DSs in hippocampal neurons. The number of AMPAR-and NMDAR-labeled gold particles at the membrane was not statistically different in hippocampal CA1 and DG regions, but the total number of NMDARs at the membrane was statistically significantly decreased in hippocampal mushroom-type DSs of early postnatal mice after 1850 MHz RF-EMF exposure for 4 weeks (Figure 2B). In this regard, a reduction in the number of active NMDA receptors may lead to synaptic dysfunction and memory decline in early postnatal mice following RF-EMF exposure.

BDNF facilitates dendritic growth and increases synapse density during dendrite development [26,27,28]. BDNF is also activated at glutamate synapses in response to activity and increases the surface expression of AMPA receptors by inducing their rapid surface translocation to increase excitatory transmission [42]. Additionally, BDNF and its receptor TrkB are involved in NMDA receptor-dependent LTP and synapse formation via presynaptic and postsynaptic mechanisms [43]. BDNF is a neurotrophic factor that contributes to neuronal survival, axonal branching, dendritic arborization, and synapse formation [26,27,28]. It supports the survival of existing neurons and promotes the growth and differentiation of new neurons and synapses [26,27]. Given that AMPA and NMDA receptors are implicated in the regulation of BDNF expression in cortical neurons [44,45], the reduction in BDNF levels may be attributed to a decrease in glutamate receptor expression in hippocampal neurons following RF-EMF exposure. Therefore, RF-EMF exposure may have perturbed neuronal survival, synapse formation, axonal branching, and dendritic arborization via BDNF-mediated effects, consequently impairing memory and learning.

It had been reported that synapses formed and matured in mouse hippocampal cultures on DIV 9, and synaptic density changed in a time-dependent manner [46,47]. Exposure to RF-EMF decreased the number of PSD95-positive puncta on DIV 5, 7, and 9 compared to that in control cells, indicating that RF-EMF exposure during early brain development reduced synaptic density. However, since this difference exhibited a ceiling effect, the difference in synaptic density may have occurred very early in development, and the effects were not cumulative. Therefore, a decrease in PSD95 expression in primary cultured hippocampal neurons following RF-EMF exposure may contribute to changes in the number and size of DSs and eventually cause disturbances in synapse stabilization and plasticity during synaptic development. PSD95 is exclusively located in postsynaptic neurons and is involved in anchoring synaptic proteins, such as glutamate receptors [48]. In particular, PSD95 protein is attached to the lower part of the NMDAR [48]. Thus, PSD95 and NMDAR interactions stabilize receptors at synaptic membranes [48]. In addition, the blockade of postsynaptic AMPARs and NMDARs statistically significantly reduced the capacity of new spines to express tagged PSD95 and decreased their probability of stabilization [49]. Our results suggest that decreased synaptic density may be underpinned by a decrease in glutamate receptors in hippocampal neurons of early postnatal mice following RF-EMF exposure, given that the reduction in PDS95 expression decreased receptor stability in DSs.

Xu et al. (2006) reported that the density of AMPAR- and NMDAR-positive punctae in cultured rat hippocampal neurons was not statistically decreased following RF-EMF exposure at SAR 2.4 W/kg for 15 min/day from DIV 7 to DIV 14 [50]. Our findings may have differed from their results because we used higher SAR values and longer exposure times. However, Xu et al. (2006) reported that RF-EMF exposure of cultured hippocampal neurons induced a decrease in the amplitude of AMPA miniature excitatory postsynaptic currents and a decrease in PSD95 expression [50]

Reduced BDNF expression in hippocampal neurons causes a decrease in neurite outgrowth [51]. MAP2 is a neuron-specific cytoskeletal protein that stabilizes microtubules and is involved in establishing dendritic arborization during development. MAP2 is thus enriched in dendrites and is used as a dendritic marker [52]. The decreased number of DSs and reduction in synaptic density, reduced synaptic densities, and neurite outgrowth may have attenuated synaptic inputs, which play important roles in synaptic strengthening, thereby affecting memory function [53,54]. The formation of dendrites is also tightly correlated with impaired nervous system function associated with neurodegenerative diseases, such as autism, depression, schizophrenia, and Alzheimer’s disease [55,56]. The hippocampus plays a crucial role in the formation of new memories and spatial navigation and is also involved in emotion and learning [9,10,11]. The hippocampus is necessary for nonspatial object memory in mice. Indeed, the novel object recognition test is associated with increased hippocampal activity encoding object identity and location [57]. Given that synaptic densities and neurite outgrowth were statistically significantly decreased following RF-EMF exposure, we assessed hippocampal-dependent memory function using the novel object recognition test [58]. The decreased synaptic densities and neurite outgrowth following RF-EMF exposure during early developmental stages may affect brain functions, specifically memory. These results are consistent with previous reports that 1.8 GHz RF-EMF exposure in C57BL/6 mice for 8 weeks caused transient impairments in spatial and nonspatial memory. Further, 2450 MHz RF-EMF-exposed rats exhibited spatial memory deficits in a water maze behavioral task [59,60].

Although the number of children using cell phones is increasing in childhood, which is the stage of neuronal development, the amount of use is also increasing. To avoid possible health hazards, ICNIRP provides the following standards for human safety protection for a normal user when exposed to electromagnetic fields of 100 kH to 300 GHz: whole body, 0.08 W/kg, head/trunk 2 W/kg, limbs 4 W/kg. In this study, pups and dams received whole-body exposure to 1850 MHz RF-EMF at a SAR value of 4.0 W/kg for 5 h per day for 4 weeks (from P1 to P28). These exposure conditions were much higher than the safety standards set by the ICNIRP guidelines [61]. Therefore, the results obtained through this experiment should not be applied to what happens to real human users. Collectively, our findings indicate that RF-EMF exposure during early brain development may decrease synaptic densities and functional synapse formation in the hippocampus by decreasing the number of DSs and expression levels of glutamate receptors and BDNF, and attenuation of neurite outgrowth. These events may result in impairments in hippocampal-dependent memory function. Therefore, our data strongly suggest that RF-EMF exposure may inhibit the development of neuronal synapse formation in the young brain and affect physiological functions.

## 4. Materials and Methods

### 4.1. Animals

ICR pups (postnatal day (P) 0) and dams were purchased from Samtako BioKorea (Osan, South Korea). Mice were maintained under specifically controlled conditions (ambient temperature, 23 ± 2 °C; 12-h light/dark cycle). Pups were fed breast milk from their mothers, which were supplied with food pellets and water ad libitum. All procedures complied with the National Institutes of Health guidelines for animal research and were approved by the Dankook University Institutional Animal Care and Use Committee (IACUC; DKU-15-001, April 14, 2015), which adheres to the guidelines issued by the Institution of Laboratory of Animal Resources.

Postnatal ICR pups were usually about 11–15, and they were exposed to RF-EMF with their mothers for 3 weeks. After 3 weeks of feeding, dams were separated from the pups and continued to be exposed to RF-EMF for a week. To prevent killing the pups by their mother, we avoided touching the pups directly and did not stress the dams by providing enough food pellets and water. The number of pups was randomly matched, and the same number of pups were provided to each dam to minimize the weight difference of the pup between each group. Litters were not gender-balanced. Furthermore, since pups were in young infancy, it was considered that there would be no difference experimentally, and all experiments were conducted without gender.

### 4.2. RF-EMF Exposure in Mice

Mice were exposed to RF-EMF using a Wave Exposer V20 (Figure S3) described in Supplementary Materials. Briefly, RF-EMF exposure was a top horn antenna (Figure S2) to the lower mouse cage. The bottom and wall of the cage were covered by ceramic wave absorption material. The mice were not restricted in movement in the cage during the exposure. All the experiments were done in our animal facility, which was maintained at constant temperature. It was confirmed that RF-EMF generator created 1850 MHz signal by a measuring spectrum analyzer (NS-30A) (LIG Nex, Gyeonggi-do, South Korea). Subsequently, the SAR value was estimated to be 4.0W/kg by a 0.0001 °C resolution temperature sensor by measuring temperature changes of saline water of the mouse phantom exposed to 1850 MHz of MHz of continuous wave (CW) without modulation. The temperature change of saline water was measured by a 0.0001 °C resolution in this research to obtain a more precise SAR value with a finer temperature measurement system (FLUKE 1586A) (Figure S4). The SAR value in the central position of the mouse phantom was also acquired by numerical analysis by Ansys HFSS 13. In addition, SAR was evaluated by measuring the E-field at the phantom position in the air and by considering the ratio of the E-field in the liquid to E-field in the air at the same position in the same environment. Our measurement of RF signal and SAR value generated from our RF-EMF generator produced 1850 MHz RF-EMF with 4.0 W/kg SAR.

Pups and dams received whole-body exposure to 1850 MHz RF-EMF at a SAR value of 4.0 W/kg for 5 h per day for 4 weeks (from P1 to P28). The sham-exposed group was maintained under the same environmental conditions and treated with the same circular pattern as that of the RF-exposed group without RF-EMF exposure. When the RF-EMF exposure group moved to the exposure device located in the animal breeding facility, the control group also moved to the same room in the animal breeding facility and returned to the original breeding room at the same time after exposure to RF-EMF. Sham- and RF-EMF-exposed mice were allowed to move freely in their cages. After the 4-week exposure, mice of either sex were sacrificed for morphological and biochemical studies. Additionally, the experiment was not carried out with a double-blind protocol, but our research results were carried out on an objective and reliable scientific basis.

### 4.3. Primary Cultures of Mouse Hippocampal Neurons

Mouse hippocampal neurons were prepared from P1 ICR mouse brains. Hippocampi were placed in Ca^2+^- and Mg^2+^-free HEPES-buffered Hanks salt solution (HHSS) (pH 7.45). Isolated tissues were incubated for 15 min in HHSS solution containing 0.025% trypsin and suspended in HHSS solution. Cells were dissociated by trituration through a 5-mL pipette and flame-narrowed Pasteur pipette. Dissociated cells were plated at a density of 1.1–105 cells/well onto an 18-mm-round cover glass in neurobasal medium with L-glutamine, 2% B27 supplement, 0.25% Glutamax I, and penicillin/streptomycin/amphotericin B (100 U/mL, 100 µg/mL, and 0.025 µg/mL, respectively). The cover glass was pre-coated with Matrigel (0.2 mg/mL; BD Bioscience, San Jose, CA, USA). Neurons were grown in a humidified atmosphere of 10% CO_2_ and 90% air at 37°C and fed on the culture day. Fresh media was used to replace 75% of the media. Prepared hippocampal neurons were exposed to 1760 MHz RF-EMF at a SAR value of 4.0 W/kg for 5 h daily for 9 days. Each experiment was performed in 3 independent cultures.

### 4.4. RF-EMF Exposure in Cultured Neurons

For RF-EMF exposure of primary hippocampal neuronal cultures, cells in culture dishes were exposed to RF-EMF radiation at 1760 MHz at a SAR value of 4.0 W/kg for 5 h daily for 9 days. The generation of this RF-EMF has been described in detail by Choi et al. (2020), who employed an RF-EMF radiation system [62]. During the exposure, the temperature of the incubator was maintained at 37  ±  0.5 °C by circulating water within the cavity, and the chamber was maintained at 5% CO_2_. The RF-EMF exposure device was installed in a CO_2_ incubator, so the non-exposed sample was in another CO_2_ incubator simultaneously. After RF-EMF exposure, both RF-EMF exposed cells and non-exposed cells were sent back to a storage CO_2_ incubator at the same time. In addition, the experiment was not performed with a double-blind protocol, but our research results were performed on an objective and reliable scientific basis.

### 4.5. Transmission Electron Microscopy (TEM)

Hippocampi obtained from sham- and RF-EMF-exposed mouse brains were fixed in Karnovsky fixative (EMS Microscopy Academy, Hatfield, PA, USA) for 4 h at 4 °C, washed three times with 0.1 M phosphate-buffered saline (PBS), and post-fixed with 1% OsO_4_ in 0.1 M PBS for 2 h at 4 °C. After washing with 0.1 M PBS, specimens were dehydrated through a graded 70–100% ethanol series, exchanged with propylene oxide, and embedded in a mixture of Epon 812 and Araldite (Polysciences Inc., Warrington, PA, USA). Ultrathin sections (70 nm) were cut using a Leica Em UC6 Ultramicrotome. A ribbon of ultrathin serial sections from each animal was collected on a Ni grid and stained with uranyl acetate and lead citrate. The sections were collected on TEM nickel grids and observed using a transmission electron microscope (JEM- 1400 flash; JEOL, Tokyo, Japan) at 120 kV.

### 4.6. Immunogold Staining

For immunogold electron microscopy (EM), sections were stained with anti-NMDAR1 (NR1), anti-AMPAR1 (GluR1), and anti-BDNF antibodies (Abcam, Cambridge, UK) diluted 1:5 in PBS containing 0.25% BSA and 0.25% gelatin for 1 h at 25 °C. After washing, sections were incubated for 1 h at 25 °C with a secondary antibody (anti-rabbit IgG-conjugated 10-nm gold particles; Electron Microscopy Science). The sections were then counterstained with uranyl acetate.

### 4.7. Immunocytochemistry

Hippocampal neurons were exposed to 1760 MHz RF-EMF for 5 h/day from 0 to 9 days in vitro (DIV). Hippocampal neurons were prepared as previously described [63]. Hippocampal neurons were fixed with cooled methanol for 10 min at −20 °C and permeabilized with 0.3% Triton X-100 for 5 min. The cells were blocked with 10% BSA and incubated for 16 h at 4 °C with the following primary antibodies: mouse anti-MAP2 (Sigma-Aldrich, St. Louis, MI, USA), rabbit anti-PSD95 (Abcam, Cambridge, UK), rabbit anti-AMPAR1 (GluR1) (Abcam, Cambridge, UK), rabbit anti-NMDAR1 (NR1) (Abcam, Cambridge, UK), and rabbit anti-BDNF (Abcam, Cambridge, UK). The cells were then incubated in Alexa Fluor 488-conjugated anti-rabbit IgG (ThermoFisher, Rockford, IL, USA) and Alexa Fluor 555-conjugated anti-mouse IgG (ThermoFisher, Rockford, IL, USA) for 1 h 30 min at room temperature. After washing in PBS, coverslips were mounted with VECTASHIELD Mounting Medium (Vector Laboratories Inc., Burlingame, CA, USA). Alexa Fluor 488 (excitation, 488 nm; emission, 520 nm) and Alexa Fluor 555 (excitation, 561 nm; emission, 568 nm)-labeled neurons were imaged using an FV3000 confocal microscope (FV3000, Olympus, Tokyo, Japan).

### 4.8. Western Blot

Sham-exposed or RF-EMF-exposed mice were rapidly sacrificed, and hippocampi were rapidly dissected. The tissue was lysed with RIPA buffer (ThermoFisher, Rockford, IL, USA) supplemented with protease and phosphate inhibitor cocktail (ThermoFisher, Rockford, IL, USA). Whole lysates were homogenized in ice-cold buffer and briefly sonicated. Protein concentrations were measured using a Bio-Rad DCTM protein assay (Bio-Rad, Hercules, CA, USA). Total protein (20–50 μg) was separated using 10–15% sodium dodecyl sulfate-polyacrylamide gel electrophoresis (SDS-PAGE) and transferred with transfer buffer to a polyvinylidene difluoride (PVDF) transfer membrane (Bio-Rad, Hercules, CA, USA). AMPAR1, NMDAR1, BDNF, and α-tubulin were detected in the membranes using anti-glutamate receptor 1 (AMPAR1/ GluR1) antibody (Abcam, Cambridge, UK), anti-NMDAR1 (NR1) antibody (Abcam, Cambridge, UK), anti-BDNF antibody (Abcam, Cambridge, UK), and anti-α-tubulin (Santa Cruz Biotechnology, Santa Cruz, CA, USA), respectively. Protein bands were visualized using an Odyssey infrared imaging system (Li-Cor Biosciences, Lincoln, NE, USA). The intensity of each band was quantified and normalized using α-tubulin as an internal loading control.

### 4.9. Cellular Imaging and Morphological Analysis

Neurons were transferred to the stage of a confocal microscope (FV3000, Olympus, Tokyo, Japan) and observed with 20× and 60× objective lenses. For the 20× objective lens, a plane optical section was captured. For the 60× objective lens, multiple optical sections spanning 7 µm in the z-dimension were collected (1-µm steps), and these optical sections were combined through the z-axis into a compressed z-stack [63]. Three images from randomly selected fields per each coverslip from 3 different cultures were taken. The counting of PSD95 puncta was performed in primary dendrites within proximal dendrites (<60 μm from soma) [64]. An algorithm was created using ImageJ software, as described previously [65], to quantify PSD95-positive puncta. Briefly, maximum z-projection images were created from MAP2 and PSD95 image stacks. Then, a threshold set of one standard deviation above the image mean was applied to the MAP2 image. This generated a 1-bit image that was used as a mask with the PSD95 maximum z-projection. The number of synaptic sites labeled with PSD95 is presented as the mean ± SEM, where *n* is the number of coverslips. The number of synaptic receptors labeled with NR1 and GluR1 is presented as the mean ± SEM, where *n* is the number of cells, each from a separate glass coverslip over multiple cultures. ImageJ software was used to analyze the intensity of staining for BDNF. Maximum z-projection images were generated from MAP2 and BDNF image stacks. The threshold values were set in each image as mean ± 4 standard deviations. MAP2 images obtained with confocal microscopy were analyzed using Metamorph software (Molecular Devices, CA, USA) to evaluate morphological changes in neurons.

### 4.10. Novel Object Recognition Test

Mice were exposed to 1850 MHz RF-EMF at a SAR value of 4.0 W/kg for 5 h per day for 4 weeks (from P1 to P28). Both sham-exposed (control) and RF-EMF-exposed mice were acclimated to the test room for 30 min. After adaptation, two objects of the same shape and size were placed in the test apparatus. The dimensions of the square apparatus were 50 cm × 50 cm (length × width). Two objects were placed at a distance of 30 cm. Time spent exploring and interacting with objects was measured for 10 min. Interaction time was defined as the time when the face and forelimbs of mice were within a 1.5 cm radius of the object. Upon conclusion of testing, mice were returned to their home cages. An hour later, one object was changed to a novel object of a different shape. Object positions were alternated between left and right sides to prevent bias in direction and position. Test mice were placed in the test apparatus, and time spent interacting with the novel object was measured for 10 min. The memory index of mice was calculated according to the formula below based on measured interaction time (in seconds).

Memory index = [(interaction time with novel object)/(interaction time with all objects)] × 100

The discrimination ratio was calculated by measuring the exploration time for the novel object compared to the old object as a proportion of the total exploration time [66]. The DR followed the formula below.

Discrimination ratio = [(interaction time with novel object) − (interaction time with old objects)]/total exploration time

### 4.11. Statistical Analysis

All data are presented as means ± SEM. All statistical analyses were performed using GraphPad Prism (GraphPad Software, La Jolla, CA, USA). Comparisons between the two groups were performed using Student’s *t*-test. One-way analysis of variance (ANOVA) was used for comparisons of data from experiments to observe changes over time in cultured hippocampal neurons. *p* < 0.05 was considered statistically significant. The *n* value represents the number of experimental samples performed independently.

## Data Availability

Not applicable.

## Supplementary Materials

The following are available online at https://www.mdpi.com/article/10.3390/ijms22105340/s1, Figure S1: Different types of dendritic spines, Figure S2: Schematic diagram of the horn antenna, Figure S3: Radiofrequency exposure apparatus (Wave Exposer V20), Figure S4: Temperature of saline water following 1850 MHz RF-EMF exposure.

## Appendix A

**Table A1:** The effect size (Cohen’s d) corresponding to each figure.

Measure	Control		RF-EMF		*p*	Cohen’s d
	M	SD	M	SD		
Figure 1Bb	3.59	2.21	2.36	1.01	0.0391	0.71
Figure 1Cb	3.19	1.78	1.64	0.67	0.0093	1.03
Figure 1Ca	2.93	1.79	1.77	0.86	0.0027	0.77
Figure 2Cg	8.33	3.77	4.08	3.20	0.0070	1.21
Figure 3Ca	42.00	13.58	17.25	5.62	0.0117	2.27
Figure 3Cb	100.00	55.18	12.00	4.55	0.0191	2.25
Figure 3Cc	67.78	46.57	14.63	5.50	0.0060	1.55
Figure 8A	61.30	8.14	54.17	15.55	0.0389	0.58
Figure 8B	0.23	0.16	0.083	0.311	0.0389	0.58

M, mean; SD, standard deviation; *p*, *p* value.

## References

[1] Christ A., Gosselin M.-C., Christopoulou M., Kühn S., Kuster N. (2010). Age-dependent tissue-specific exposure of cell phone users. Phys. Med. Biol..

[2] Kim J.H., Sohn U.D., Kim H.G., Kim H.R. (2018). Exposure to 835 MHz RF-EMF decreases the expression of calcium channels, inhibits apoptosis, but induces autophagy in the mouse hippocampus. Korean J. Physiol. Pharmacol..

[3] Kim J.H., Yu D.H., Huh Y.H., Lee E.H., Kim H.G., Kim H.R. (2017). Long-term exposure to 835 MHz RF-EMF induces hyperactivity, autophagy and demyelination in the cortical neurons of mice. Sci. Rep..

[4] Kim J.H., Lee C.H., Kim H.G., Kim H.R. (2019). Decreased dopamine in striatum and difficult locomotor recovery from MPTP insult after exposure to radiofrequency electromagnetic fields. Sci. Rep..

[5] Birks L.E., Struchen B., Eeftens M., van Wel L., Huss A., Gajšek P., Kheifets L., Gallastegi M., Dalmau-Bueno A., Estarlich M. (2018). Spatial and temporal variability of personal environmental exposure to radio frequency electromagnetic fields in children in Europe. Environ. Int..

[6] Foster K.R., Chou C.-K. (2014). Are Children More Exposed to Radio Frequency Energy From Mobile Phones Than Adults?. IEEE Access.

[7] Gandhi O.P., Morgan L.L., De Salles A.A., Han Y.-Y., Herberman R.B., Davis D.L. (2011). Exposure Limits: The underestimation of absorbed cell phone radiation, especially in children. Electromagn. Biol. Med..

[8] Kim J.H., Huh Y.H., Lee J.-H., Jung J.Y., Ahn S.C., Kim H.R. (2019). Early exposure to radiofrequency electromagnetic fields at 1850 MHz affects auditory circuits in early postnatal mice. Sci. Rep..

[9] Konkel A., Cohen N.J. (2009). Relational memory and the hippocampus: Representations and methods. Front. Neurosci..

[10] Deng W., Aimone J.B., Gage F.H. (2010). New neurons and new memories: How does adult hippocampal neurogenesis affect learning and memory?. Nat. Rev. Neurosci..

[11] Eichenbaum H., Cohen N.J. (2014). Can We Reconcile the Declarative Memory and Spatial Navigation Views on Hippocampal Function?. Neuron.

[12] Wang L., Zang Y., He Y., Liang M., Zhang X., Tian L., Wu T., Jiang T., Li K. (2006). Changes in hippocampal connectivity in the early stages of Alzheimer’s disease: Evidence from resting state fMRI. Neuroimage.

[13] Del Campo M., Hoozemans J.J., Dekkers L.-L., Rozemuller A.J., Korth C., Müller-Schiffmann A., Scheltens P., Blankenstein M.A., Jimenez C.R., Veerhuis R. (2014). BRI2-BRICHOS is increased in human amyloid plaques in early stages of Alzheimer’s disease. Neurobiol. Aging.

[14] Campbell S., MacQueen G. (2004). The role of the hippocampus in the pathophysiology of major depression. J. Psychiatr. Neurosci..

[15] Videbech P., Ravnkilde B. (2004). Hippocampal volume and depression: A meta-analysis of MRI studies. Am. J. Psychiatr..

[16] Morris R.G.M., Moser E.I., Riedel G., Martin S.J., Sandin J., Day M., O’Carroll C. (2003). Elements of a neurobiological theory of the hippocampus: The role of activity-dependent synaptic plasticity in memory. Philos. Trans. R. Soc. B Biol. Sci..

[17] Robinson P.J. (2007). How to fill a synapse. Science.

[18] Kim J.H., Kim H.-J., Yu D.-H., Kweon H.-S., Huh Y.H., Kim H.R. (2017). Changes in numbers and size of synaptic vesicles of cortical neurons induced by exposure to 835 MHz radiofrequency-electromagnetic field. PLoS ONE.

[19] Kim J.H., Lee J.-K., Kim H.-G., Kim K.-B., Kim H.R. (2019). Possible Effects of Radiofrequency Electromagnetic Field Exposure on Central Nerve System. Biomol. Ther..

[20] Kim J.H., Huh Y.H., Kim H.R. (2019). Trafficking of synaptic vesicles is changed at the hypothalamus by exposure to an 835 MHz radiofrequency electromagnetic field. Gen. Physiol. Biophys..

[21] Südhof T.C. (2004). The synaptic vesicle cycle. Annu. Rev. Neurosci..

[22] Xu X., Miller E.C., Pozzo-Miller L. (2014). Dendritic spine dysgenesis in Rett syndrome. Front. Neuroanat..

[23] Bourne J., Harris K.M. (2007). Do thin spines learn to be mushroom spines that remember?. Curr. Opin. Neurobiol..

[24] Newpher T.M., Ehlers M.D. (2008). Glutamate Receptor Dynamics in Dendritic Microdomains. Neuron.

[25] Debanne D., Daoudal G., Sourdet V., Russier M. (2003). Brain plasticity and ion channels. J. Physiol..

[26] Sanchez A.L., Matthews B.J., Meynard M.M., Hu B., Javed S., Cohen-Cory S. (2006). BDNF increases synapse density in dendrites of developing tectal neurons in vivo. Development.

[27] Koshimizu H., Kiyosue K., Hara T., Hazama S., Suzuki S., Uegaki K., Nagappan G., Zaitsev E., Hirokawa T., Tatsu Y. (2009). Multiple functions of precursor BDNF to CNS neurons: Negative regulation of neurite growth, spine formation and cell survival. Mol. Brain.

[28] Harward S.C., Hedrick N.G., Hall C.E., Parra-Bueno P., Milner T.A., Pan E., Laviv T., Hempstead B.L., Yasuda R., McNamara J.O. (2016). Autocrine BDNF-TrkB signalling within a single dendritic spine. Nature.

[29] Von Bohlen und Halbach O., von Bohlen und Halbach V. (2018). BDNF effects on dendritic spine morphology and hippocampal function. Cell Tissue Res..

[30] Holmes J.R., Berkowitz A. (2014). Dendritic orientation and branching distinguish a class of multifunctional turtle spinal interneurons. Front. Neural Circuits.

[31] Southam K.A., Stennard F., Pavez C., Small D.H. (2019). Knockout of amyloid β protein precursor (APP) expression alters synaptogenesis, neurite branching and axonal morphology of hippocampal neurons. Neurochem. Res..

[32] Huang E.J., Reichardt L.F. (2001). Neurotrophins: Roles in Neuronal Development and Function. Annu. Rev. Neurosci..

[33] Hering H., Sheng M. (2001). Dentritic spines: Structure, dynamics and regulation. Nat. Rev. Neurosci..

[34] Schikorski T., Stevens C.F. (1997). Quantitative Ultrastructural Analysis of Hippocampal Excitatory Synapses. J. Neurosci..

[35] Nusser Z., Lujan R., Laube G., Roberts J.B., Molnar E., Somogyi P. (1998). Cell Type and Pathway Dependence of Synaptic AMPA Receptor Number and Variability in the Hippocampus. Neuron.

[36] Xiong J., He C., Li C., Tan G., Li J., Yu Z., Hu Z., Chen F. (2013). Changes of Dendritic Spine Density and Morphology in the Superficial Layers of the Medial Entorhinal Cortex Induced by Extremely Low-Frequency Magnetic Field Exposure. PLoS ONE.

[37] Lang C., Barco A., Zablow L., Kandel E.R., Siegelbaum S.A., Zakharenko S.S. (2004). Transient expansion of synaptically connected dendritic spines upon induction of hippocampal long-term potentiation. Proc. Nat. Acad. Sci. USA.

[38] Matsuzaki M., Honkura N., Ellis-Davies G.C.R., Kasai H. (2004). Structural basis of long-term potentiation in single dendritic spines. Nat. Cell Biol..

[39] McEntee W.J., Crook T.H. (1993). Glutamate: Its role in learning, memory, and the aging brain. Psychopharmacology.

[40] Henley J.M., Wilkinson K.A. (2013). AMPA receptor trafficking and the mechanisms underlying synaptic plasticity and cognitive aging. Dialog. Clin. Neurosci..

[41] Takumi Y., Ramírez-León V., Laake P., Rinvik E., Ottersen O.P. (1999). Different modes of expression of AMPA and NMDA receptors in hippocampal synapses. Nat. Neurosci..

[42] Cunha C., Brambilla R., Thomas K.L. (2010). A simple role for BDNF in learning and memory?. Front. Mol. Neurosci..

[43] Yoshii A., Constantine-Paton M. (2007). BDNF induces transport of PSD-95 to dendrites through PI3K-AKT signaling after NMDA receptor activation. Nat. Neurosci..

[44] Xiong H., Futamura T., Jourdi H., Zhou H., Takei N., Diverse-Pierluissi M., Plevy S., Nawa H. (2002). Neurotrophins induce BDNF expression through the glutamate receptor pathway in neocortical neurons. Neuropharmacology.

[45] Zheng F., Wang H. (2009). NMDA-mediated and self-induced bdnf exon IV transcriptions are differentially regulated in cultured cortical neurons. Neurochem. Int..

[46] Dotti C.G., Sullivan C.A., Banker G.A. (1988). The establishment of polarity by hippocampal neurons in culture. J. Neurosci..

[47] Virdee J.K., Saro G., Fouillet A., Findlay J., Ferreira F., Eversden S., O’Neill M.J., Wolak J., Ursu D. (2017). A high-throughput model for investigating neuronal function and synaptic transmission in cultured neuronal networks. Sci. Rep..

[48] El-Husseini A.E.-D., Schnell E., Chetkovich D.M., Nicoll R.A., Bredt D.S. (2000). PSD-95 involvement in maturation of excitatory synapses. Science.

[49] De Roo M., Klauser P., Mendez P., Poglia L., Muller D. (2007). Activity-Dependent PSD Formation and Stabilization of Newly Formed Spines in Hippocampal Slice Cultures. Cereb. Cortex.

[50] Xu S., Ning W., Xu Z., Zhou S., Chiang H., Luo J. (2006). Chronic exposure to GSM 1800-MHz microwaves reduces excitatory synaptic activity in cultured hippocampal neurons. Neurosci. Lett..

[51] Almeida M.F., Chaves R.S., Silva C.M., Chaves J.C., Melo K.P., Ferrari M.F. (2016). BDNF trafficking and signaling impairment during early neurodegeneration is prevented by moderate physical activity. IBRO Rep..

[52] Harada A., Teng J., Takei Y., Oguchi K., Hirokawa N. (2002). MAP2 is required for dendrite elongation, PKA anchoring in dendrites, and proper PKA signal transduction. J. Cell Biol..

[53] Mozzachiodi R., Byrne J.H. (2010). More than synaptic plasticity: Role of nonsynaptic plasticity in learning and memory. Trends Neurosci..

[54] Raven F., Van der Zee E.A., Meerlo P., Havekes R. (2018). The role of sleep in regulating structural plasticity and synaptic strength: Implications for memory and cognitive function. Sleep Med. Rev..

[55] Tavosanis G. (2011). Dendritic structural plasticity. Dev. Neurobiol..

[56] Penzes P., Cahill M.E., Jones K.A., VanLeeuwen J.-E., Woolfrey K.M. (2011). Dendritic spine pathology in neuropsychiatric disorders. Nat. Neurosci..

[57] Cohen S.J., Munchow A.H., Rios L.M., Zhang G., Ásgeirsdóttir H.N., Stackman R.W. (2013). The Rodent Hippocampus Is Essential for Nonspatial Object Memory. Curr. Biol..

[58] Götz J., Ittner L.M. (2008). Animal models of Alzheimer’s disease and frontotemporal dementia. Nat. Rev. Neurosci..

[59] Ntzouni M.P., Skouroliakou A., Kostomitsopoulos N., Margaritis L.H. (2012). Transient and cumulative memory impairments induced by GSM 1.8 GHz cell phone signal in a mouse model. Electromagn. Biol. Med..

[60] Wang C., Lai J. (2000). Modelling the vibration behaviour of infinite structures by FEM. J. Sound Vib..

[61] International Commission on Non-Ionizing Radiation Protection (ICNIRP) (2020). Guidelines for Limiting Exposure to Electromagnetic Fields (100 kHz to 300 GHz). Health Phys..

[62] Choi J., Min K., Jeon S., Kim N., Pack J.-K., Song K. (2020). Continuous Exposure to 1.7 GHz LTE Electromagnetic Fields Increases Intracellular Reactive Oxygen Species to Decrease Human Cell Proliferation and Induce Senescence. Sci. Rep..

[63] Kim H.J., Martemyanov K.A., Thayer S.A. (2008). Human immunodeficiency virus protein Tat induces synapse loss via a reversible process that is distinct from cell death. J. Neurosci..

[64] Baj G., Patrizio A., Montalbano A., Sciancalepore M., Tongiorgi E. (2014). Developmental and maintenance defects in Rett syndrome neurons identified by a new mouse staging system in vitro. Front. Cell. Neurosci..

[65] Waataja J.J., Kim H.J., Roloff A.M., Thayer S.A. (2008). Excitotoxic loss of post-synaptic sites is distinct temporally and mechanistically from neuronal death. J. Neurochem..

[66] Sivakumaran M.H., MacKenzie A.K., Callan I.R., Ainge J.A., O’Connor A.R. (2018). The Discrimination Ratio derived from Novel Object Recognition tasks as a Measure of Recognition Memory Sensitivity, not Bias. Sci. Rep..

