# Supplementary material for: Exposure to RF-EMF Alters Postsynaptic Structure and Hinders Neurite Outgrowth in Developing Hippocampal Neurons of Early Postnatal Mice
Source: Int J Mol Sci. 2021 May 19;22(10):5340. doi: 10.3390/ijms22105340 (PMC8159076; doi:10.3390/ijms22105340)
Supplement: Supplementary file 1 [file ijms-22-05340-s001.zip › ijms-1148911-supplementary.pdf]

Supplementary Materials

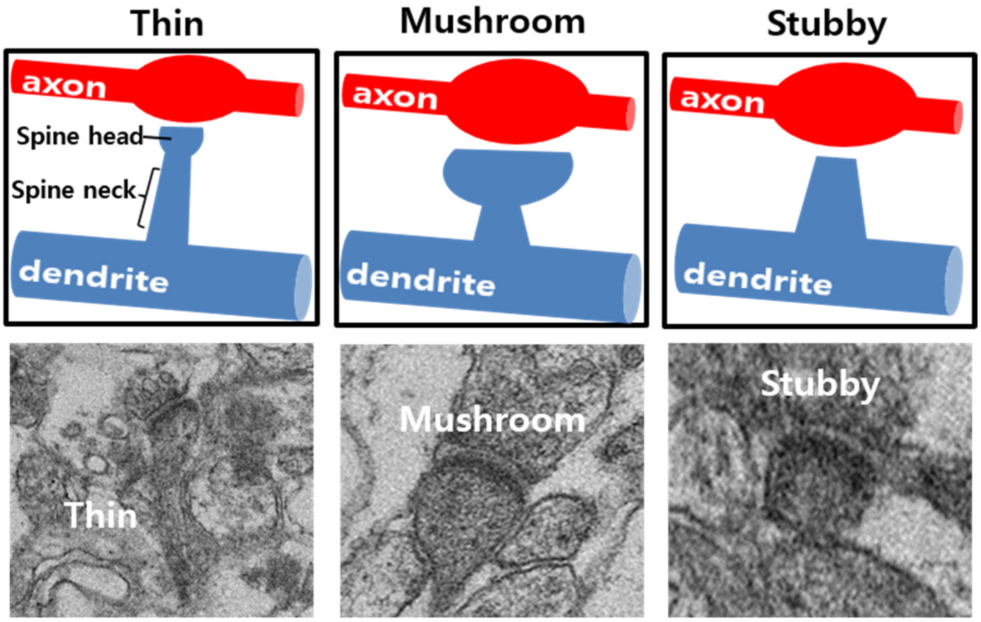

Figure S1. Different types of dendritic spines. Schematic diagrams of spine morphologies based on the most common three-category classification: thin, mushroom, and stubby spines (upper panel). Network of neurites (postsynaptic spines) with dendritic spines and two spines contacting axons (presynaptic boutons). Representative images of different types of dendritic spines in mouse hippocampal neurons observed using TEM (lower panel).

SAR measurement in a liquid mouse phantom exposed by 1850 MHz rectangular horn antenna system.

RF exposure system (Figure S2, S3) was generated to investigate the effect of radiation on mouse at 1850 MHz of continuous wave (CW) without modulation. Firstly, we estimated RF signal with spectrum analyzer (NS-30A) (LIG Nex, Gyeonggi-do, South Korea). We confirm that the RF-EMF instrument generator generates 1850 MHz RF-EMF signals. Subsequently, SAR value was estimated to be 4.0W/kg by 0.0001˚C resolution temperature sensor by measuring temperature changes of saline water of the mouse phantom exposed to 1850 MHz of CW. Temperature change of saline water was measured by 0.0001℃ resolution in this research to obtain more precise SAR value with a finer temperature measurement system. SAR value in the central position of the mouse phantom was also acquired by numerical analysis by Ansys HFSS 13. Also, SAR was evaluated by measuring E-field at the phantom position in the air and by considering the ratio of E-field in the liquid to E-field in the air at the same position in the same environment.

RF power (-11.7 dBm) was generated, amplified (43 dB) and driven to the waveguide to coaxial adapter through 1.2 meter long cable (1.3 dB loss). It was eventually radiated by a pyramidal rectangular horn antenna of 37 cm × 27 cm aperture after waveguide to coaxial adapter.

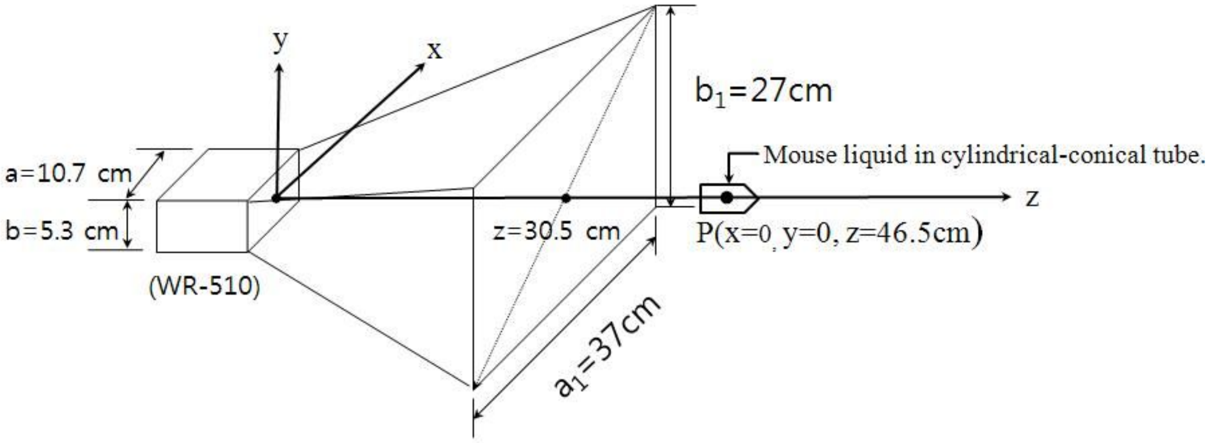

Figure S2. **Schematic diagram of the horn antenna.**

SAR (specific absorption rate) at a point in the lossy medium exposed by high frequency electromagnetic field is defined in eq. (1). (IEEE Std 1528TM-2013, IEEE Recommended Practice for Determining the Peak Spatial-Average Specific Absorption Rate (SAR) in the Human Head from Wireless Communications Devices: Measurement Techniques, IEEE Standards Coordinating Committee 39, IEEE).

$$SAR = C\frac{dT}{dt}\bigg|_{t=0} = \frac{\sigma|E|^2{}_{mag}}{2\varrho} \text{ [W/kg]} \qquad (1)$$

Specific heat capacity of the saline water C is 4,140 [J/kg ˚C], while electrical conductivity of the medium σ was 2.044 [S/m]. mag is electric field intensity in magnitude of phasor[V/m], mass density ϱ of 0.9 % saline water is 1,002 [kg/m3] at 20˚C, but it is set to be 1,000 [kg/m3] in usual SAR measurements. T is temperature (˚C) of 0.9 % saline water, and t is duration time of 1850 MHz RF exposure. The changes of saline water's temperature (˚C) following different exposure time of 1850 MHz RF showed in Figure S4. SAR value was calculated by the conditions that ΔT = 0.6˚C and Δt = 1200 sec. SAR = 4.02W/kg in this case.

Following calculation, the value is 4.02W/kg which is proximity of setting up output power at SAR 4.0 W/kg of horn antenna of the exposure apparatus. As a result, our measurement of RF signal and SAR value generated from our RF generator produce 1850 MHz RF-EMF with 4.0 W/kg SAR.

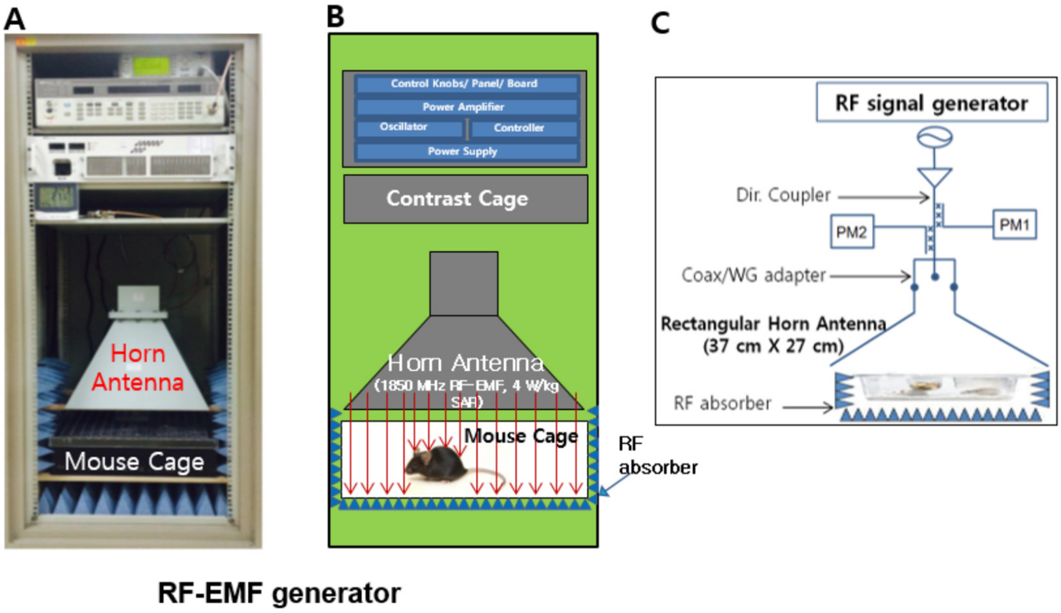

Figure S3. **Radiofrequency exposure apparatus (Wave Exposer V20)** is used for RF-EMF exposure of mice to 1,850MHz at 4.0W/kg SAR. (A) An exterior view of RF generator. (B) Schematic outline in RF generator. (C) System block diagram of horn antenna and mouse cage.

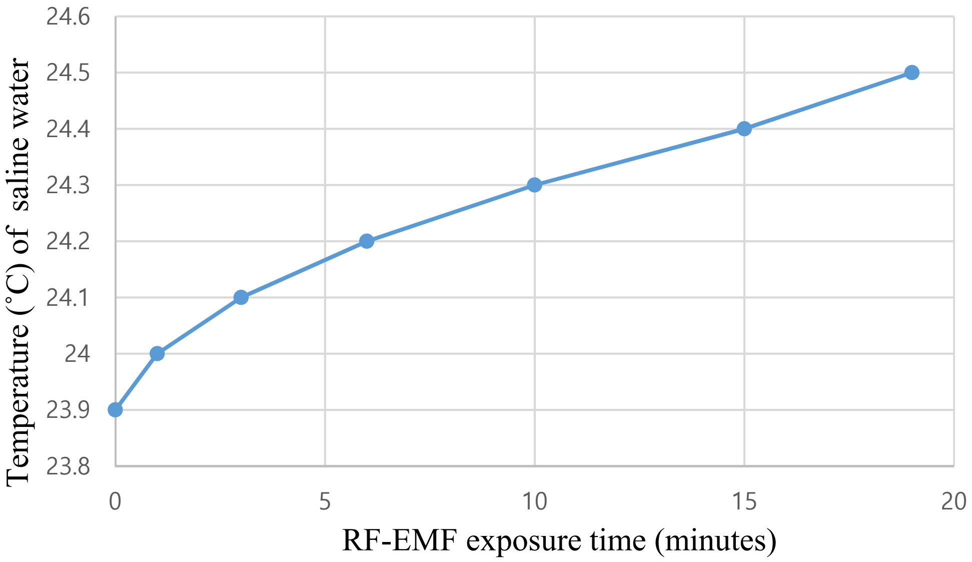

Figure S4. Temperature of saline water following 1,850 MHz RF-EMF exposure. Change of 0.9 % saline water's temperature (˚C) following deferent exposure time of 1850 MHz RF-EMF by RF generator. Room temperature was 23.9-24.1˚C.
